# Drainless Uniportal VATS Wedge Resection for Early Non-Small Cell Lung Cancer: Propensity Analysis of the Effect of Polyglycolic Acid Sheet (Neoveil^TM^)

**DOI:** 10.3390/diagnostics14222586

**Published:** 2024-11-18

**Authors:** Shuenn-Wen Kuo, Yu-Heng Su, Ke-Cheng Chen

**Affiliations:** Division of Thoracic Surgery, Department of Surgery, College of Medicine, National Taiwan University Hospital and National Taiwan University, Taipei 100, Taiwan; shuenn8@gmail.com (S.-W.K.); victorysu@gmail.com (Y.-H.S.)

**Keywords:** Neoveil, drainless VATS, early non-small cell lung cancer, surgical outcome, PGA, wedge resection

## Abstract

**Objectives:** Absorbable biomaterials as adjuvant therapy after thoracoscopy are sometimes used in clinical scenarios. With the prevalence of enhanced rapid recovery in thoracic surgery, drainless video-assisted thoracoscopy surgery (VATS) is often adopted by thoracic surgeons. Here, we discuss utilizing an absorbable biomaterial, Neoveil^TM^ (Polyglycolic Acid sheet), for drainless VATS to treat early lung cancer. **Methods:** This single-center retrospective study was conducted from January 2018 to December 2022 at the National Taiwan University Hospital. We included patients who underwent drainless VATS for early-stage non-small cell lung cancer (NSCLC) in our institute. Propensity analysis was used to minimize selection bias. Outcome measurements were in-hospital stay, operation time, rate of thoracocentesis or chest drain re-insertion, complication rate, and perioperative course. **Results:** During the study period, 158 lung cancer patients were performed with drainless VATS wedge resection. Among them, Neoveil for stapling line coverage was done in 72 patients, while 86 patients did not receive Neoveil. After propensity analysis, we had 58 patients using Neoveil after drainless thoracoscopic lung resection, compared fairly with 58 patients without Neoveil after the same procedure. The basic characteristics are comparable regarding age, gender, BMI, operation methods, and lung cancer stage after propensity matching. The in-hospital stay (3.2 days in the Neoveil group and 5.6 days in the non-Neoveil group) and operation time (95.7 min in the Neoveil group and 59.3 min in the non-Neoveil group) are significantly different (*p* = 0.0001). One versus four patients was noted for postoperative conversion chest drainage insertion in each group (*p* = 0.17). Neither late complications nor recurrence/metastasis occurred in both groups during the following. **Conclusions:** Based on our 5-year retrospective study, which is balanced with propensity analysis, drainless thoracoscopic surgery treating early lung cancer can be enhanced by Neoveil with faster recovery by reducing the hospital stay, though with longer operation time.

## 1. Introduction

Lung cancer has become the most devastating cancer worldwide [1]. Low-dose computed tomography (LDCT) is a promising screening tool for early diagnosis. The NELSON study confirmed the efficacy of LDCT screening for lung cancer while showing its value in decreasing mortality [2]. LDCT screening can detect many lung cancer patients in the early stage. Since then, appropriate surgical resection for early non-small cell lung cancer (NSCLC) based on minimally invasive surgery with video-assisted thoracoscopic surgery (VATS) has become increasingly popular and important. However, safety and enhanced rapid recovery (ERAS) under the VATS remains challenging. Postoperative lung parenchymal air leakage and bleeding of the resection wound were noted sometimes [3].

**Figure 1:**
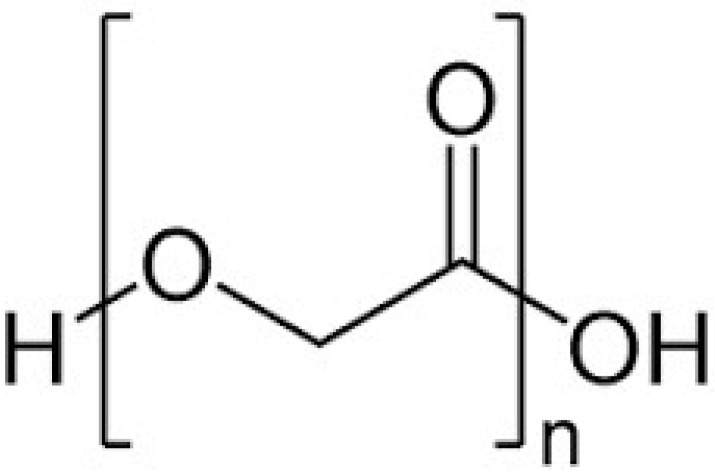
The molecular structure of Neoveil^TM^.

Recently, using biomaterial polymer mesh to cover the resection wound is more and more common during surgery. Neoveil^TM^ (GUNZE, Kyoto, Japan) is a bioabsorbable, biocompatible soft-tissue reinforcement material derived from 100% polyglycolic acid (Figure 1). It is used as an adjuvant for lung parenchyma to prevent air leakage and enhance hemostasis and tissue healing [3]. Since the wide acceptance of LDCT screening, sublobar resection is an appropriate procedure for ground glass opacity (GGO)-dominant early NSCLC. The clinical application of ERAS has also become popular and important in lung cancer surgery. There is no difference in short-term and long-term survival outcomes between sublobar resection and lobectomy for early and peripheral lung cancer [4,5,6]. VATS wedge resection, as one of the sublobar resection methods, can preserve more lung volume than lobectomy. Even more, VATS for wedge resection without drain is feasible [7]. It is the so-called drainless VATS surgery. It can improve the perioperative course, rapid recovery, cosmetics, and satisfaction. However, pneumothorax or pleural effusion was sometimes noted, which may require thoracocentesis or salvage chest drain [7]. The utilization of the Neoveil started and was proven decades ago in Japan, but the earliest English literature was in 2005, when it was applied in spine surgery [8]. Applying an absorbable biomaterial sheet to the stapling line of a resected lung can enhance healing and hemostasis while preventing air leakage in lung surgery [3]. Herein, we analyze the feasibility and surgical outcome of a Neoveil-covered wedge resection stapling line after resection of small pulmonary lesions in this retrospective study. The main objective of this study was to report the experience of adjuvant usage with Neoveil^TM^ (Polyglycolic Acid) after drainless thoracoscopic lung cancer resection, compared with the same procedure without Neoveil.

## 2. Materials and Methods

### 2.1. Study Population

This was a single-center retrospective study from January 2018 to December 2022, using a data bank from the National Taiwan University Hospital, a 3500-bed medical center in East Asia. We included patients who underwent uniportal thoracoscopic lung surgery and selected those who received drainless VATS by authors (Dr. Kuo and Chen) in our group. The Research Ethics Committee of the National Taiwan University Hospital approved this study. During the study period, 1493 patients in our group received uniportal VATS surgery. The exclusion criteria include missing data, patients undergoing open surgery, and patients with postoperative drains. Among them, 158 patients underwent drainless uniportal VATS. Overall, 72 patients were scheduled to receive adjuvant Neoveil Sheet stapling line covering after uniportal drainless VATS. Eighty-six patients underwent regular drainless VATS without Neoveil usage.

### 2.2. Technique for Drainless Uniportal VATS Wedge Resection

**Figure 2:**
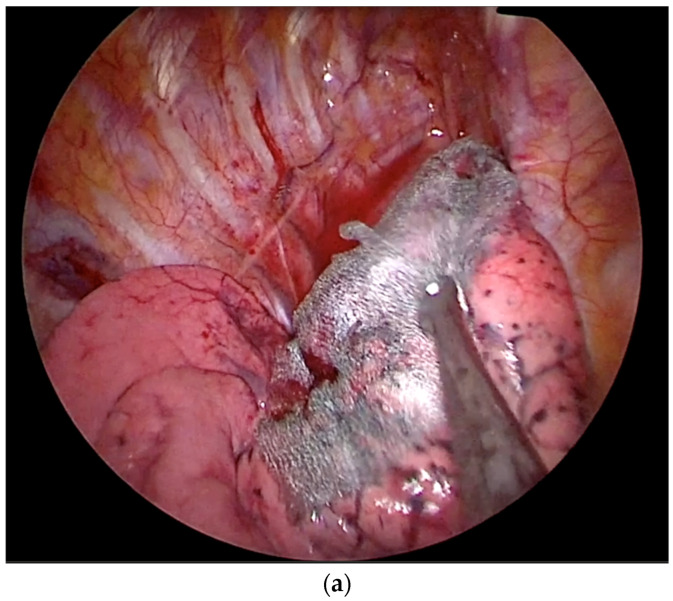

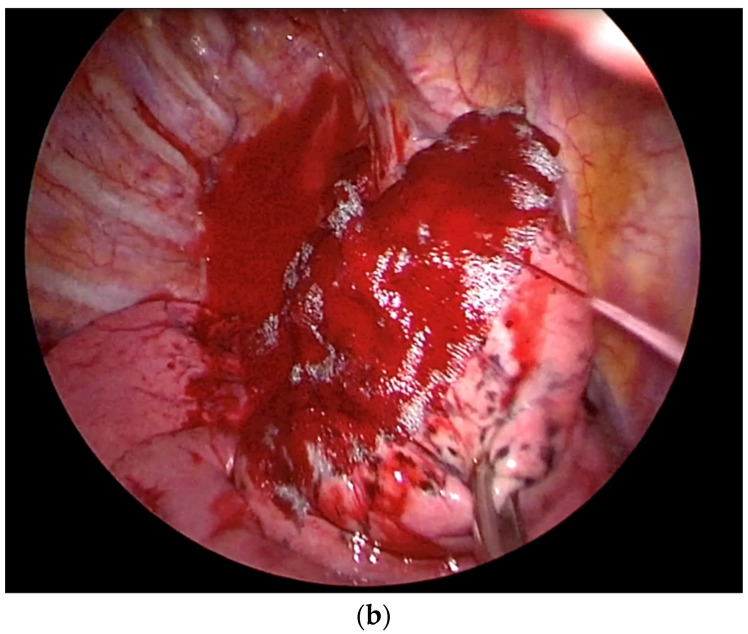
(**a**) The Neoveil sheet was placed on the cutting line of the lung. (**b**) Pouring autologous blood drawn from the patient on the surface of Neoveil.

Preoperatively, patients received blood testing, including a hemogram, liver/kidney function, electrolytes, and lung function test by Jaeger spirometry. The patient was placed in the lateral decubitus position, and the ipsilateral lung was deflated under general anesthesia and intubation using a double-lumen endotracheal tube or a single-lumen tube with a blocker. A 2-cm single skin incision was performed at the anterior axillary line along the 5th or 6th intercostal space, and a wound protector was applied at the incision site. A 5-mm, 30-degree video telescope (Karl Storz, Tuttlingen, Germany or Olympus, Tokyo, Japan) was used during surgery. Through the single wound, the location of lung tumors was noted by finger palpation and (or) instrument touching or by Patent Blue V dye localization (Guerbet, Aulnay-sous-Bois, France) under preoperative computed tomography. An endo-GIA stapler was used for wedge resection. For the regular wedge resection group without Neoveil usage, we removed all the air within the chest, closed the wounds in layers without draining after resection, and checked for air leakage. On the other hand, for those in the Neoveil group, we applied a 5.0 cm × 5.0 cm Neoveil sheet on the stapling line surface, followed by pouring 10 mL autologous blood drawing from the patient (Figure 2). Afterward, we removed the air and closed the wound without placing any drain tubes. The above air-removing method was as follows: using a silicone CWV drain tube (seven mm) connected to a vacuum ball (CWV reservoir, 150 mL). The drainage tube was connected to a vacuum ball outside the chest cavity, and the CWV catheter was placed to remove residual air and pleural effusion before wound closure. The whole procedure was shown in our supplementary video (Video S1).

**Figure 3:**
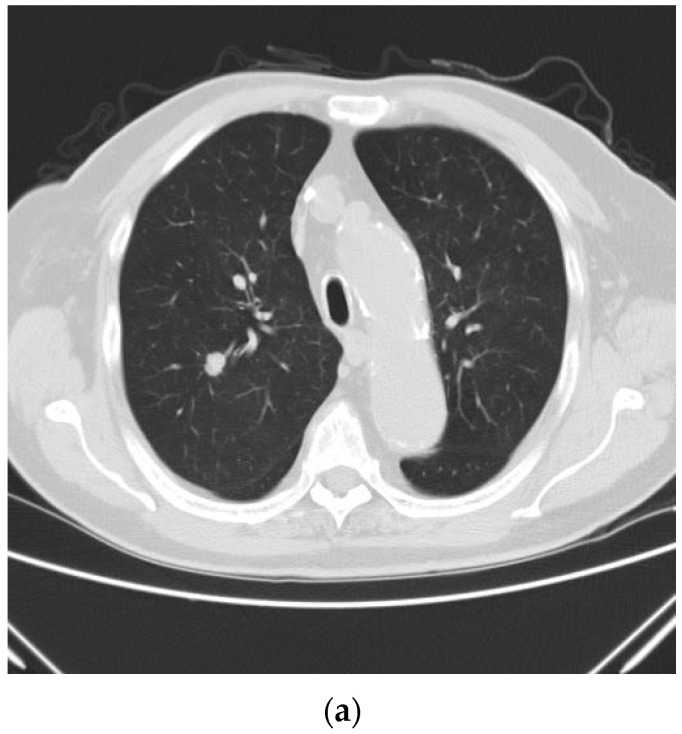

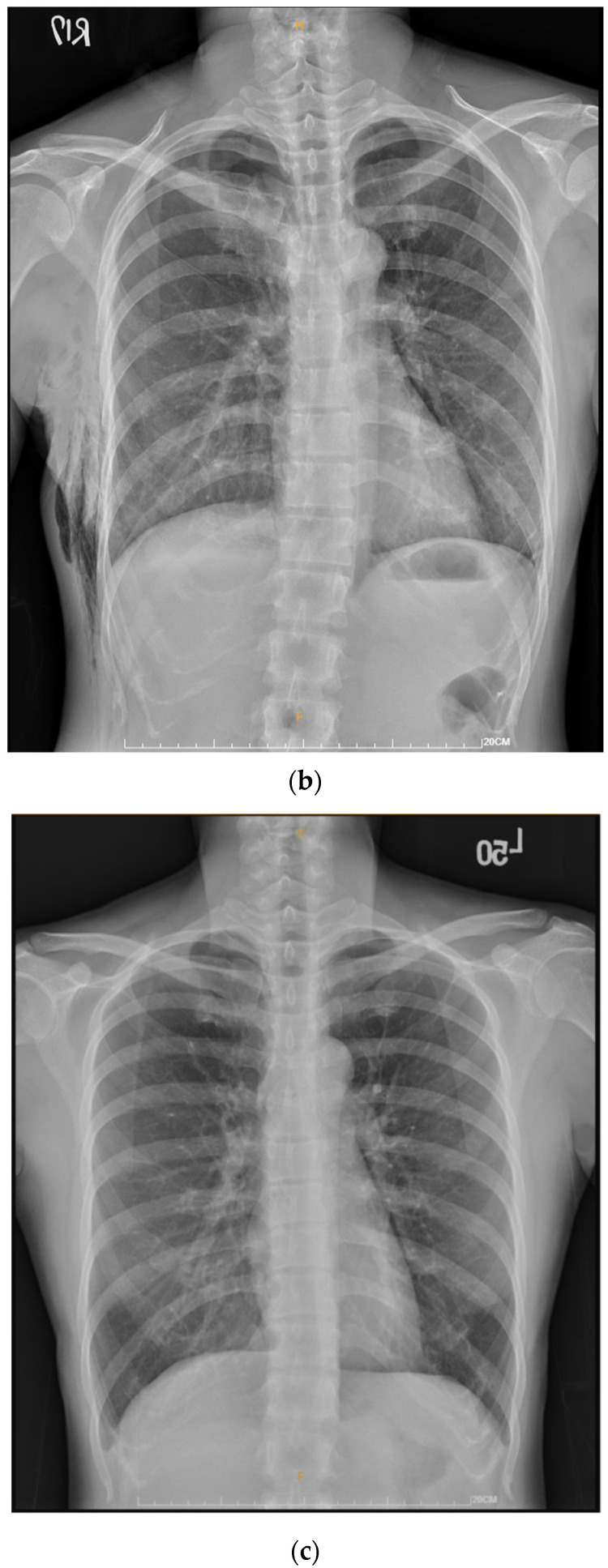
(**a**) Preoperative CT showed a lung tumor in the right upper lung. (**b**) The postoperative chest plain film was taken on the operation day immediately after the surgery. Some subcutaneous emphysema was noted on the right side. (**c**) The chest plain film on the day of the first outpatient return after discharge. No detectable air within the body was noted.

The patients were able to resume oral intake within hours postoperatively. Postoperative pain control was provided by regular oral nonsteroidal analgesics or acetaminophen. Patient-controlled analgesia with morphine (1 mg/mL) was administered intravenously as required by the patients. A chest X-ray was performed on the operation day (Figure 3). Thoracocentesis or pleural drainage tube insertion was performed if a serial chest X-ray showed progress of pneumothorax in a 24-h postoperative period. Patients were ready to be discharged if chest X-ray showed no progressive pneumothorax. The size of residual pneumothorax was defined as the largest distance between the pleural line and the chest wall on the chest plain film. Patients were eligible for discharge if no notable pneumothorax (less than five cm in diameter) was noted on serial chest plain film. If the chest plain film revealed considerable pneumothorax (five cm or more in diameter) or progress of the residual pneumothorax or if the patient’s respiratory status deteriorated clinically, a chest tube would be inserted, or needle aspiration would be performed. The first outpatient follow-up was on postoperative day 10, when the follow-up chest X-ray was taken (Figure 3).

### 2.3. Statistical Analysis

Outcome measurements in this study were in-hospital stay, operation time, rate of thoracocentesis or chest drain re-insertion, complication rate, and perioperative course of thoracoscopic drainless VATS for early lung cancer. We estimated propensity scores and matched them using an eighth-to-first-digit greedy matching algorithm to create a cohort of matched patients with comparable observed characteristics. The propensity score was calculated by logistic regression, which included the lesion number, lesion depth, tumor size, tumor location, and patient underlying with lung emphysema or chronic obstructive pulmonary disease with pulmonary function. Patients with similar propensity scores were assigned to the same group. The sample size ratio is 1:1 for the two groups. All values in this study were presented as mean ± standard deviation. We used IBM SPSS version 21.0 to analyze the data (IBM Co., Armonk, NY, USA), and all statistical tests were two-sided. The unpaired *t*-test, chi-square test, and Fisher’s exact test were used to evaluate between-group differences. All *p*-values less than 0.05 were regarded as statistical significance.

## 3. Results

### 3.1. Patient Demographics

During the study period, 158 patients (46 men and 112 women) with early-stage NSCLC undergoing drainless uniportal VATS wedge resection were included. The mean age was 51.7 ± 9.8 years, and the median body mass index (BMI) was 23.3 ± 3.1 (kg/m^2^). The median pulmonary function test showed forced expiratory volume in one second (FEV1) as 108.1 ± 21.5% and functional vital capacity (FVC) as 109.4 ± 13.6%. There were 132 patients in ASA class I, 24 patients in ASA class II, two patients in ASA class III, and zero patients in ASA class IV. After propensity analysis, two matching groups were selected with a 1:1 ratio. The results showed they are comparable regarding age, gender, BMI, underlying diseases, blood testing results, lung function test, nodule size, depth, and location (Table 1).

### 3.2. Postoperative Outcomes

The hospital stay (3.2 days in the Neoveil group and 5.6 days in the non-Neoveil group) and operation time (95.7 min in the Neoveil group and 59.3 min in the non-Neoveil group) were significantly different (*p* = 0.0001). The postoperative pneumothorax rate was 12.1% (Neoveil group) versus 19.0% (non-Neoveil group), which showed no significant difference (*p* = 0.31). One case of mild hemothorax required conservative treatment in the non-Neoveil group. One out of four patients was noted for postoperative conversion chest tapping or drainage tube insertion in each group (*p* = 0.17) (Table 2). There were no Neoveil-related complications or adverse effects in the study group, and safety margins for cancer were satisfactory in both groups. There were no late complications, and all patients were cancer-free in both groups during postoperative follow-up.

## 4. Discussion

Lung cancer is a major health threat globally [1]. Low-dose computed tomography (LDCT) can detect lung cancer at early stages, for which a minimally invasive surgical procedure using VATS is the best strategy. Previously, lobectomy was required to resect early lung cancer. Currently, a minor procedure, including wedge resection, may be enough for selected cases [4,5,6]. Meanwhile, many novel methods have been invented recently to assist in accurate localization and resection [9]. By way of this, thoracic surgeons can easily identify the lesion. After identifying the lesion, we use an endo-GIA stapler to resect the lung tumor with margins. Due to the advanced technology, the staplers are very reliable and of good quality. Therefore, omitting the pleural drainage tubes was possible. However, postoperative pneumothorax or pleural effusion occurred. Drainless uniportal VATS for early lung cancer is a good method for selected patients [7]. It can be used in early lung cancer presented as peripheral pulmonary lung nodules and has the advantage of rapid recovery. However, sometimes it fails because of air leakage or pleural effusion. Moreover, the hospital stay is prolonged if postoperative pneumothorax occurs. Few studies discussed the methods employed to cover the stapling line after resection of small lung tumors with bioabsorbable mesh. Previous applications of bioabsorbable biomaterials mainly focused on pneumothorax, and the effect was satisfactory [10,11]. In our study, postoperative pneumothorax was noted in either group. Some patients required salvage therapy with thoracocentesis or pleural pigtail drainage [12]. Therefore, preventing pneumothorax or pleural effusion can make drainless VATS even more successful. Neoveil™ (polyglycolic acid mesh sheet) is a tissue-strengthening repair agent that prevents air or fluid leakage after surgery [3]. The application of the Neoveil sheet was approved and started decades ago in Japan [3,8,10,11]. The preventive effect of Neoveil has been proven in various surgical fields; for example, these sheets reduce the pancreatic fistula incidence in pancreas resection [13,14,15]. Moreover, liver surgery and head/neck surgery are also used to enhance the result [16,17,18]. All the results showed satisfactory outcomes, and there were no major side effects attributed to the biocompatible nature and leakage-preventing effects. In lung surgery, besides pneumothorax, Neoveil has been successfully adopted to treat postoperative bronchial stump fistula after lobectomy [19]. Although the cutting line of wedge resection by an endo-GIA stapler is quite reliable, sometimes trouble happens. Previously, it was unclear whether using Neoveil helps in uniportal VATS wedge resection without placing a pleural drain. In the current study, we applied a Neoveil sheet, followed by autologous blood after uniportal VATS lung cancer wedge resection. The texture of Neoveil is very soft and conforming, with good biocompatibility and tissue affinity. Our study showed good clinical outcomes and is the first study in the world. Nonetheless, the absorbable biomaterial Neoveil is not cheap and costs about 1000 USD in our institute. National health insurance does not cover it, and patients who need it must pay for themselves. Moreover, the operation time is significantly prolonged, which is disadvantageous for old and weak patients. In our current study, most patients could tolerate the surgery safely and had shorter in-hospital stays with peaceful clinical courses. Outpatient surgery is possible for those early lung cancers presented as solitary peripheral lung tumors. The tremendous number of early lung cancer patients in Asia makes the beds in the ward unavailable. If drainless uniportal VATS with Neoveil sheet works, we can make outpatient thoracic surgery even more common. Thus, thoracic surgeons can handle more patients screened by large-scale, low-dose CT lung cancer screening.

Salvage thoracocentesis for drainless VATS in our study was 4.3%. The Non-Neoveil group had a rate of 6.9%, while the Neoveil group had a rate of 1.7%, comparable with the previous study [12]. For most patients, a small amount of residual air and pleural effusion will absorb itself in a few days. However, persistent air leakage, small vessel bleeding, and lymphatic vessel leakage can cause trouble. While we must improve our surgical skills, proper usage of adjuvant biomaterial products is important, too. Rapid recovery was established based on safety, especially in thoracic surgery [20]. Notably, our group successfully operated on patients using the non-intubated technique, i.e., non-intubated VATS [21]. Thus, combining drainless VATS with non-intubation anesthesia without foley can produce so-called tubeless VATS. Tubeless VATS causes minimal discomfort and is the first step of outpatient surgery, the ultimate goal of lung cancer surgery.

This study was limited by its retrospective design and small number of patients, though under the balancing of propensity analysis. Meanwhile, short-term follow-up makes it difficult to differentiate the specific benefits of further oncologic outcomes. Therefore, further studies with a larger number of patients and long-term follow-ups are needed to confirm or dismiss our results.

In conclusion, in drainless VATS wedge resection of early lung cancer, Neoveil can aid ERAS by reducing in-hospital stays. Meanwhile, we proved it as a feasible way of accomplishing this goal by reducing the need for salvage chest drainage.

## Figures and Tables

**Table 1:** Clinical characteristics and demographics of two matched groups.

Variables	Neoveil Group (*n* = 58) Number (%), or Mean ± SD (Range)	Non-Neoveil Group (*n* = 58) Number (%), or Mean ± SD (Range)	*p*-Value
Age, year	52.5 ± 10.9 (38~71)	48.5 ± 11.2 (36~73)	0.574
Male	22 (37.9%)	24 (41.4%)	0.152
Ever smoker	14 (24.1%)	16 (27.6%)	0.704
Height, cm	161.9 ± 6.9 (148.0–178.0)	162.8 ± 7.9 (151.0–178.5)	0.681
Weight, kg	60.8 ± 7.5 (49.0–76.5)	61.0 ± 8.0 (51.0–78.0)	0.754
FVC (L)	2.8 ± 0.7	2.9 ± 0.6	0.631
FVC (%)	110.8 ± 15.8	108.6 ± 12.1	0.613
FEV1 (L)	2.4 ± 0.6	2.3 ± 0.5	0.370
Hb	13.2 ± 1.4	13.4 ± 1.6	0.840
CRP	0.45 ± 0.15	0.47 ± 0.20	0.544
Fasting glucose	95.0 ± 16.0	94.5 ± 15.5	0.865
COPD/Emphysematous lung	5 (8.6%)	4 (6.9)	0.729
DM	7 (12.1%)	6 (10.3%)	0.769
Heart disease	5 (8.6%)	8 (13.8%)	0.377
GGO/Solid nodule	38/20	36/22	0.699
Nodule number			
1	48 (40%)	48 (40.0%)	1.000
2	10 (60%)	10 (60.0%)	
Nodule size, cm	1.1 ± 0.6 (0.5–1.8)	1.0 ± 0.5 (0.6–1.7)	0.881
Nodule depth, cm	1.9 ± 0.5 (1.5–2.7)	1.8 ± 0.4 (1.4–2.6)	0.909
Ratio of size/depth	56.1 ± 16.9% (22.2–84.9%)	61.9 ± 14.8% (22.7–89.5%)	0.195
Location			NS
Right	26 (44.8%)	28 (48.3%)	
Left	32 (55.2%)	30 (51.7%)	
Upper	22 (37.9%)	20 (34.5%)	

NS = Non-significant.

**Table 2:** The outcome of drainless uniportal VATS wedge resection with and without Neoveil sheet coverage.

Variables	Neoveil Group (*n* = 58) Number (%), or Mean ± SD (Range)	Non-Neoveil Group (*n* = 58) Number (%), or Mean ± SD (Range)	*p*-Value
Surgery time (min)	95.7 ± 11.5 (62–127)	59.3 ± 9.3 (32–86)	0.0001
Chest tube drainage (days)	0	0	NS
Hospital stay (days)	3.2 ± 0.8 (2–5)	5.6 ± 0.9 (2–8)	0.0001
Other complications	0 (0%)	0 (0%)	NS
Pneumothorax (Discharge)	7 (12.1%)	11 (19.0%)	0.31
Pneumothorax (first Outpatient)	3 (5.2%)	4 (6.9%)	0.70
Hemothorax	0 (0%)	1 (1.7%)	NS
Conversion to thoracocentesis or drain insertion	1 (1.7%)	4 (6.9%)	0.17
Margin involved	0 (0%)	0 (0%)	NS

NS = Non-significant.

## Data Availability

The data underlying this article will be shared on reasonable request to the corresponding author.

## Supplementary Materials

The following supporting information can be downloaded at: https://www.mdpi.com/article/10.3390/diagnostics14222586/s1, Video S1: Drainless uniportal VATS wedge resection followed by Neoveil usage.

## Abbreviations

VATS	Video-Assisted Thoracoscopic Surgery
LDCT	Low dose computed tomography
NSCLC	Non-small Cell Lung Cancer
GGO	Ground glass opacity
